# Epidemiological features and specificities of HCV infection: a hospital-based cohort study in a university medical center of Calabria region

**DOI:** 10.1186/1471-2334-12-S2-S4

**Published:** 2012-11-12

**Authors:** MC Liberto, N Marascio, E Zicca, G Matera

**Affiliations:** 1 Department of Health Sciences, Institute of Microbiology, University “Magna Graecia” of Catanzaro, 88100, Catanzaro, Italy

## Abstract

The epidemiological status of HCV in Europe, and in particular in Mediterranean countries, is continuously evolving. The genotype distribution is related to improvement of healthcare conditions, expansion of intravenous drug use and immigration. We review and characterize the epidemiology of the distribution of HCV genotypes within Calabria, an area of Southern Italy. We focus on the pattern of distinct HCV genotype changes over the last 16 years; particularly subtype 1b and genotype 4. We collected data by evaluating a hospital-based cohort of chronic hepatitis C patients; in addition, we report an update including new patients enrolled during last eight months.

## Introduction

Hepatitis C virus (HCV) triggers worldwide chronic liver diseases, as cirrhosis and hepatocellular carcinoma (HCC). HCV infection represents the most important cause of liver transplantation in developed countries.

Despite the decline of the viral transmission rates, due to eradication of transfusion-associated infections and improvement in health-care-related standards, HCV-related morbidity and mortality continue to rise and, due to slow disease progression, many patients are still at risk to progress to severe liver diseases [1]. For this reason prevention of HCV infections as well as improved knowledge of HCV infection epidemiology are particularly noteworthy.

HCV genotyping is an important approach in the management of HCV RNA positive patients and it is useful to understand the epidemiology of the virus with respect to the demographic characteristics of patients, liver disease grade, risk factors and source of infection. Genotyping has also been used to found HCV geographical distribution: subtype 1b is spreading worldwide, subtype 3a is clustering in Europe and North America and genotype 4 appears endemic in Middle East and Central Africa. It is already known that the prevalence of HCV, in a particular area, can be unstable and HCV changing pattern occurs because of several factors, as high rates of mutation in viral genome, drug pressure and migration of infected people to countries where a more sensitive population is established.

On this HCV changing pattern, surveillance studies have been carried out in several European regions [2,3].

Since 1997, our group studied distribution of HCV genotypes within Calabria, a region of Southern Italy, by evaluating a hospital-based cohort of chronic hepatitis C patients. This mini-review summarizes published data on the epidemiology of HCV within this area, focusing on the pattern of distinct HCV genotype changes over the last 16 years, particularly subtype 1b and genotype 4. In addition, an update of the last eight months will be addressed.

## HCV spread in the Mediterranean countries of Europe

In Europe, the epidemiology of HCV infection is rapidly changing and prevalence, as well as risk factors and genotype distribution exhibit significant geographic and temporal differences. In Northern and Central Europe the HCV prevalence is ranging from 0.2% (in the Netherlands) to 1.2% (in France) [2], virus is mainly transmitted by intravenous drug use (IDU) and the most prevalent infections are found among patients 30–50 years old. In countries of the Mediterranean area of Europe the overall prevalence ranges between 2.5% and 3.5% [2], spread via blood products as well as IDU-related infections have been determined. Most importantly, immigration is leading to changes in HCV epidemiology and in the distribution of HCV genotypes. In Spain HCV prevalence is among the highest in Europe, with high rate of genotype 3 (mainly among IDUs) while the increasing rate of genotype 4 has been reported to be linked to IDU and immigration [4,5].

Differences in prevalence of HCV infection have been observed in Turkey, where higher rates are reported in regions near the Black Sea when compared with other areas; the genotype 1b was ranging between 75 and 90% in chronic infected patients [1,6,7]. In Turkey, the non-hospitalized childbirth or invasive and poor-hygiene standard medical procedures may increase the risk of transmission of HCV [1]. In Greece, another country in the Mediterranean region of Europe, the prevalence of the disease is high (6–10%) in some isolated areas and it is mainly due to unsafe parenteral injections or invasive medical procedures. Genotype 1 is predominant among chronically infected people, genotype 3 is rising and 15% of infections are caused by genotype 4 [8,9].

In Italy, the HCV epidemiology has some specificity. In a HCV seroprevalence and molecular epidemiology study, performed in a sample of the Italian general population, Ansaldi et al., [10] showed a north–south prevalence gradient and confirmed that Central and Southern Italy are hyper endemic areas. In this study HCV subtype 1b was the most prevalent in all geographical areas followed by subtypes 2c, 4a/4d, 3a and 1a. The prevalence of subtype 1b increased with age.

Overall, risk factors are related to hospital-based transmission, healthcare-related or IDU transmission and, concerning age distribution, a high prevalence of HCV in the elderly has been established. Different studies reported on the genotype distribution of HCV in Italy [11-15]. Genotype 1b appeared to be the most frequent type of infection [13-15] followed by genotype 2, which is more common in Italy than in other parts of Europe. Likewise, in a small epidemiological study, carried out in a Southern Italian town, HCV subtype 2a has been reported to be predominant with respect to subtype 1b [11]. Changes in the prevalence of HCV genotypes among Italian injection drug users, in relation to the time of infection, have been reported [12], namely a decline in genotype 3 has been observed, while genotype 4 has become more frequent. The prevalence of HCV type 4 increased in Southern [16], and Central Italy [14]. Genotype 5 and 6 HCV were rarely found as well as mixed infections with more genotypes [1].

## HCV in Calabria region: epidemiological features

In our first investigation we report data on changes in the prevalence of hepatitis C virus genotype 4 in Calabria, Southern Italy. In particular we evaluated prevalence of circulating HCV genotypes, as well as relationships between genotype, sex and age in sera from 3273 HCV-positive patients collected from January 1997 until February 2001 [16].

The main findings on genotype distribution showed that HCV 1b was the most common subtype (1744 cases, 53.3%) followed by 2a/2c (857 cases, 26.2%) and both were associated with older age. This distribution was similar to other studies regarding the prevalence of HCV genotypes in Italy and Western Europe.

Genotype 4 was the fifth most prevalent genotype observed, just after 3a and 1a subtypes. Analyzing the distribution of the HCV genotype 4 during three consecutive periods of 17, 19 and 13 months, within the full observation period, we reported an early increase (January 1997–May 1998) of the genotype 4 (3.3% versus 1.3%),in comparison to prevalence findings previously observed by Guadagnino et al., [17]. Data obtained from serum samples, collected from June 1998 until December 1999 and from January 2000–February 2001, indicated a further increase in prevalence of genotype 4 (3.7% and 4.7% respectively).

Therefore, on HCV positive sera collected from January 1997 until February 2001, we verified a fourfold increased prevalence of HCV genotype 4. While in this period other authors found type 4 to be related to IDU in younger individuals [18], by contrast our data showed that spreading of genotype 4 in the Calabria region was associated with older age, when compared to genotype 3a and 1a, and associated with a younger group of patients when compared with genotype 1b. Moreover, HCV genotype 4-infected patients denied having traveled to endemic areas and most of them lived in rural areas. We assumed that such an increase could be partially due to previous undervaluation of the real prevalence of genotype 4 in the Calabria. Moreover, we hypothesized that this HCV genotype could be endemic in some restricted and isolated areas of Calabria.

Recently we updated data on the distribution of HCV genotypes, by evaluating a hospital-based cohort of 2153 chronic hepatitis C patients, collected prospectively among subjects attending University Hospital of Catanzaro, Calabria, Southern Italy [19]. The rates of HCV genotypes during two consecutive periods, from 2001 to 2005 and from 2006 to 2011, according to age and gender were considered. Concerning the eleven-year period of observation, subtype 1b was predominant (49.2%), followed by subtype 2a/2c, genotypes 3 and 4, with a rate of 22.4%, 7.4% and 6.2% respectively.

Still at present subtype 1b is the most common HCV type associated with risk factors such as blood/blood derivatives transfusion and surgical procedures. Subtype 1b-infected patients are >65 years old, accordingly with Ansaldi et al., who reported a transmission pattern found in adults older than 60 years, due to health care-related practices in the past and characterized by subtypes 1b and 2c infections [10]. As in other European reports, our data show that 1b prevalence decreased over the past eleven years and this finding is mainly due to the improved health standards that reduced HCV transmission.

When data of the two consecutive periods were examined, a higher percentage of HCV infection in >65 age group (33.7% versus 41.2%) was observed. Between 2001 and 2005, the distribution of subtype 1b was similar in 56 - 65 and >65 age groups, while between 2006 and 2011 data showed an increased distribution in >65 age group with respect to 56 - 65 age group. Interestingly, during the 2006–2011 period HCV genotype 4 was mostly present in patients 56 - 65 years old (31.6%), while two different peaks, during the 2006 – 2011 period, were observed: 27.6% among the 36 - 45 years old and 24.1% in patients over 65 years old.

Therefore, during the 2006 - 2011 period, statistical analysis of HCV patient age, stratified by genotypes, showed a slight but significant increase in the median age of subtype 1b, subtype 2a/2c and genotype 3 HCV infected subjects, while genotype 4 patients exhibited a decrease in the median age during the same period studied.

Genotype 4 is to date the fourth prevalent genotype in our area and the observation regarding the shift of the highest prevalence of genotype 4 towards younger age group (36 - 45) during 2006 - 2011, sounds as a challenge for epidemiologists. Probably this trend could be related to increased sexual risk behavior.

## Update of HCV epidemiology in Calabria

**Figure 1:**
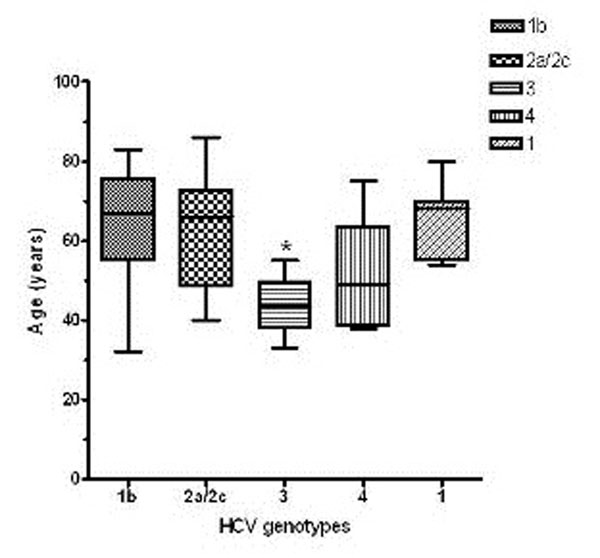
Box plot of genotypes 1b, 2a/2c, 3, 4 and 1 distribution by HCV patients age in the January-August 2012 period. _*_ p < 0.05 vs. genotype 1b.

The update of our epidemiology investigation includes the new subjects enrolled as a hospital – based cohort of HCV-infected patients during last eight months. The age of patients with genotype/subtype 3 was significantly lower than age of subjects with genotypes/subtypes 1b (p < 0.05 Tukey post-hoc test). Also the age of patients with genotype/subtype 2a/2c, 4 and 1 was higher than the age of subjects with genotype 3, without a statistical significance (Figure 1). Dealing with gender distribution of genotypes/subtypes HCV we found a significant (p < 0.05 by χ^2^ test) predominance of females among the 2a/2c –positive subjects, by contrast the male gender was significantly associated with genotype 3 (p < 0.05 by χ^2^ test). Regarding the viral load the subtypes 2a/2c and 1a shared the highest values; the genotypes/subtypes 1b, 1 and 3 exhibit the medium values, and genotypes 4 and 2 showed a lower amount of viral load (Table 1).

**Table 1:** HCV genotypes/subtypes distribution, gender and viral load of 67 patients from January to August 2012.

HCV genotypes/ subtypes	No. of isolates	Percentage (%)	Gender				P value	Log_10_ HCV-RNA (UI/ml) Mean± SEM
					
			Male	(%)	Female	(%)		
1	7	10.5	2	28.6	5	71.4	> 0.05	6.6 ± 6.2
1b	29	43.3	19	65.5	10	34.5	> 0.05	6.7 ± 6.4
1a	4	5.9	3	75.0	1	25.0	> 0.05	7.0 ± 6.6
2	1	1.5	0	0.0	1	100.0	> 0.05	3.3 ± 0.0
2a/2c	17	25.4	6	35.3	11	64.7	< 0.05	7.1 ± 6.7
3	4	5.9	4	100.0	0	0.0	< 0.05	3.7 ± 6.3
4	5	7.5	3	60.0	2	40.0	> 0.05	3.2 ± 5.9
Total	67	100	37	55.2	30	44.8		3.9 ± 6.3

## Conclusion

HCV epidemiology shows a high variability across Mediterranean countries of Europe and Italy. The evaluation of chronic HCV prevalence, genotype distribution and risk factors for transmission, represent a great challenge, which will pay off in achievement of efficient measures for hepatitis C prevention. Nation-specific strategies could be applied in different countries to decrease spreading of HCV infection. The most representative change in HCV genotype prevalence, observed in our region, has been reported to be an increase in genotype 4. Appropriated interventions are warranted to control genotype 4 epidemics because this genotype is associated with a great risks of complications (cirrhosis and HCC) [20] and a poor response to standard treatment. Such interventions can now be difficult because the ways of transmission are expanding, including not only IDU, but also, probably, sexual risk behaviors.

## List of abbreviations used

HCV: Hepatitis C Virus; IDU: Intravenous Drug Use; HCC: hepatocellular carcinoma.

## Competing interests

The authors declare that they have no competing interests.

## Declarations

Publication of this supplement was partly supported by an unrestricted grant provided by Roche. The articles were independently prepared by the authors with no input from Roche. Roche were not involved in selecting the articles for the supplement.
